# GTS-21, an α7nAChR agonist, increases pulmonary bacterial clearance in mice by restoring hyperoxia-compromised macrophage function

**DOI:** 10.1186/s10020-020-00224-9

**Published:** 2020-10-30

**Authors:** Ravikumar A. Sitapara, Alex G. Gauthier, Vivek S. Patel, Mosi Lin, Michelle Zur, Charles R. Ashby, Lin L. Mantell

**Affiliations:** 1 Department of Pharmaceutical Sciences, St. John’s University College of Pharmacy and Health Sciences, 8000 Utopia Parkway, Queens, NY 11439 USA; 2 The Feinstein Institute for Medical Research, Northwell Health System, Manhasset, NY 11030 USA

**Keywords:** α7nAChR, Hyperoxia, Macrophage function, HMGB1, NF-κB, Acute lung injury, Pulmonary infection

## Abstract

**Background:**

Mechanical ventilation, in combination with supraphysiological concentrations of oxygen (i.e., hyperoxia), is routinely used to treat patients with respiratory distress, such as COVID-19. However, prolonged exposure to hyperoxia compromises the clearance of invading pathogens by impairing macrophage phagocytosis. Previously, we have shown that the exposure of mice to hyperoxia induces the release of the nuclear protein high mobility group box-1 (HMGB1) into the pulmonary airways. Furthermore, extracellular HMGB1 impairs macrophage phagocytosis and increases the mortality of mice infected with *Pseudomonas aeruginosa* (PA). The aim of this study was to determine whether GTS-21 (3-(2,4-dimethoxybenzylidene) anabaseine), an α7 nicotinic acetylcholine receptor (α7nAChR) agonist, could (1) inhibit hyperoxia-induced HMGB1 release into the airways; (2) enhance macrophage phagocytosis and (3) increase bacterial clearance from the lungs in a mouse model of ventilator-associated pneumonia.

**Method:**

GTS-21 (0.04, 0.4, and 4 mg/kg) or saline were administered by intraperitoneal injection to mice that were exposed to hyperoxia (≥ 99% O_2_) and subsequently challenged with PA.

**Results:**

The systemic administration of 4 mg/kg i.p. of GTS-21 significantly increased bacterial clearance, decreased acute lung injury and decreased accumulation of airway HMGB1 compared to the saline control. To determine the mechanism of action of GTS-21, RAW 264.7 cells, a macrophage-like cell line, were incubated with different concentrations of GTS-21 in the presence of 95% O_2_. The phagocytic activity of macrophages was significantly increased by GTS-21 in a dose-dependent manner. In addition, GTS-21 significantly inhibited the cytoplasmic translocation and release of HMGB1 from RAW 264.7 cells and attenuated hyperoxia-induced NF-κB activation in macrophages and mouse lungs exposed to hyperoxia and infected with PA.

**Conclusions:**

Our results indicate that GTS-21 is efficacious in improving bacterial clearance and reducing acute lung injury via enhancing macrophage function by inhibiting the release of nuclear HMGB1. Therefore, the α7nAChR represents a possible pharmacological target to improve the clinical outcome of patients on ventilators by augmenting host defense against bacterial infections.

## Introduction

Oxygen therapy, using mechanical ventilation (MV) with supraphysiological concentrations of oxygen (hyperoxia), is a lifesaving intervention for patients with respiratory distress (Ruggiu et al. 2018). However, patients on ventilators become highly susceptible to lung infections and have a greater likelihood of developing ventilator-associated pneumonia (VAP). *Pseudomonas aeruginosa* (PA), a gram-negative aerobic bacterium, has been reported to be associated with 21% of all VAP cases (Liu et al. 2008; Richards et al. 1999). VAP accounts for up to 60% of all deaths from hospital-acquired infections in the United States and continues to be a major cause of morbidity and mortality in patients on ventilators (Tablan et al. 2003; Ramirez Barba et al. 2006; Davis 2006).

Alveolar macrophages (AMs) are the first-line of defense against invading pathogens and are the earliest effectors of the phagocytic response against microbial infections in the distal airways (Aberdein et al. 2013; Franke-Ullmann et al. 1996). AMs isolated from animals and cultured macrophages exposed to hyperoxia have impaired phagocytosis of pathogens such as PA and *Klebsiella pneumoniae*, as well as paraffin oil droplets (Morrow et al. 2007; Patel et al. 2013; O’Reilly et al. 2003; Baleeiro et al. 2003; Rister 1982; Raffin et al. 1980; Crowell et al. 1995). Impaired macrophage functions have been correlated with increased susceptibility and severity of bacterial infections in animals exposed to hyperoxia (Patel et al. 2013; Baleeiro et al. 2003). However, macrophage bacterial clearance functions impaired by hyperoxia can be attenuated by compounds that enhance antioxidant capacity (Patel et al. 2016, 2020), donate exogenous nitric oxide (Gore et al. 2020), or inhibit NF-κB activation (Wang et al. 2015). The resulting enhanced macrophage functions can mitigate hyperoxia-induced excessive pro-inflammatory response in the lungs of mice and decrease acute lung injury (Patel et al. 2016; Entezari et al. 2014; Sitapara et al. 2020).

Prolonged exposure to hyperoxia also induces the accumulation of high mobility group box-1 protein (HMGB1) in the airways of mice and in the media of cultured macrophages (Patel et al. 2013, 2020; Entezari et al. 2012, 2014). High levels of airway HMGB1 have been reported in patients with cystic fibrosis and in patients on ventilators (Entezari et al. 2012; van Zoelen et al. 2008). Extracellular HMGB1 in the airways is sufficient to impair the phagocytic function of AMs (Entezari et al. 2012). Furthermore, HMGB1-compromised macrophage functions can result in decreased host defenses against bacterial infection in animal models of cystic fibrosis and VAP (Patel et al. 2013; Entezari et al. 2012). Thus, decreasing the accumulation of HMGB1 in the airways of patients with CF and VAP may provide an important therapeutic strategy for these patients.

Numerous studies have been directed towards elucidating the mechanisms underlying the release of nuclear HMGB1 into the extracellular milieu in order to develop treatments or interventions that attenuate the adverse effects of extracellular HMGB1 (Yang 2005; Wang 1999). The release of HMGB1 can be partly controlled by the cholinergic anti-inflammatory reflex (Andersson and Tracey 2011; Kang et al. 2014; Wang et al. 2019). This anti-inflammatory reflex relays the presence of inflammation, danger, and pathogen-associated molecular pattern signals via the afferent vagus nerve to the central nervous system for integration (Andersson and Tracey 2011; Kang et al. 2014; Wang et al. 2019). As a result, efferent vagus nerves are activated and stimulate the release of acetylcholine from effector cells, which activates the α7 nicotinic acetylcholine receptors (α7nAChR) on cells such as macrophages to decrease the production and secretion of pro-inflammatory cytokines (Andersson and Tracey 2011; Kang et al. 2014; Wang et al. 2019). Indeed, α7nAChR activation plays a critical role in inhibiting the release of nuclear HMGB1 into the extracellular milieu (Wang et al. 2019; Ulloa 2005; Wang et al. 2004).

Macrophages express high levels of α7nAChR, which may be a target for decreasing the accumulation of extracellular HMGB1 (Wang et al. 2003; Khan et al. 2012). Indeed, α7nAChR activation plays a critical role in inhibiting LPS-induced release of nuclear HMGB1 into the extracellular milieu (Wang et al. 2019; Ulloa 2005; Wang et al. 2004). GTS-21, 3-(2,4-dimethoxybenzylidene) anabaseine, is an agonist of α7nAChR (Pavlov et al. 2007; Giebelen et al. 2007). GTS-21 has been shown to decrease the extracellular accumulation of HMGB1 in LPS-stimulated AMs (Wang et al. 2019). Furthermore, in a mouse model of LPS-induced acute lung injury, GTS-21 decreased the (1) release of HMGB1 into the airways and serum and (2) HMGB1 mRNA expression (Wang et al. 2019). In order for nuclear HMGB1 to be actively released into the serum or the airways, it first needs to be translocated from the nucleus into the cytoplasm, which is associated with the activation of NF-κB (Wang et al. 2015; Kang et al. 2014; Wang et al. 2019). HMGB1 release and its extracellular accumulation from cultured macrophages after prolonged exposure to hyperoxia is accompanied by the activation of NF-κB (Wang et al. 2015). By decreasing hyperoxia-induced activation of NF-κB, NF-κB inhibitors can inhibit both hyperoxia-induced-HMGB1 releases from macrophages and macrophage dysfunction (Wang et al. 2015). Thus, the aim of this study was to determine the effects of GTS-21 on (1) hyperoxia-reduced host defense to clear PA infection in a mouse model of VAP, (2) hyperoxia-induced suppression of macrophage phagocytosis, and (3) the accumulation of extracellular HMGB1 in the airways of animals subjected to prolonged exposure to hyperoxia and PA lung infection.

## Materials and methods

### Cell culture and reagents

Murine macrophage-like RAW 264.7 cells (American Type Culture Collection-TIB-71, Manassas, VA) were cultured in RPMI 1640 medium (GIBCO/BRL Life Technologies Inc., Grand Island, NY) supplemented with 10% fetal bovine serum (Atlanta Biologicals, Lawrenceville, GA), 1% penicillin and 1% streptomycin (Life Technologies, Grand Island, NY). The cells were maintained at 37 °C under (5% CO_2_/21% O_2_) normoxic condition for 24 h and allowed to grow to 70-80% confluency and were subcultured every three days. GTS-21, 3-(2,4-dimethoxybenzylidene)-anabaseine dihydrochloride, was obtained from Abcam (Cambridge, MA).

### Bronchoalveolar lavage

Murine bronchoalveolar lavage (BAL) fluid was obtained as described previously (Patel et al. 2013). Briefly, mice were anesthetized by an intraperitoneal injection of sodium pentobarbital (120 mg/kg). After a 1- to 2-cm incision was made on the neck, the trachea was dissected, and a 20-gauge × 1.25-inch intravenous catheter was inserted caudally into the lumen of the exposed trachea. The lungs were gently lavaged twice with 1 ml sterile, nonpyrogenic PBS solution (Mediatech, Inc., Hendon, VA). The BAL samples were centrifuged, and the resultant supernatants were stored in a freezer at − 80 °C for analyzing concentrations of extracellular HMGB1 and the total protein content using Western blot analysis and a bicinchoninic acid assay, respectively.

### Animal studies

C57BL/6 mice (male, 8–12-week-old; The Jackson Labs, Bar Harbor, ME) were used in this study in accordance with the Institutional Animal Care and Use Committee of St. John’s University (Queens, NY). The mice were housed in a specific pathogen-free environment that was maintained at 22 °C in ≈ 50% relative humidity and with a 12 h light/dark cycle. All mice had ad libitum access to standard rodent chow and water. Mice were randomized to receive either GTS-21 (0.04, 0.4, and 4 mg/kg) or saline, administered by intraperitoneal injection, every 8 h, starting 32 h after the onset of hyperoxic exposure. After 48 h of exposure, the mice were inoculated with 0.1 × 10^8^ CFU of PA by making a 1 to 2 cm incision on the neck to expose the trachea following anesthetization with sodium pentobarbital (60 mg/kg). Eighteen hours after bacterial inoculation, mice were euthanized with intraperitoneal sodium pentobarbital (120 mg/kg) to obtain BAL and lung tissues as described previously (Patel et al. 2013). After lavaging with PBS, the lungs were excised and immediately placed into 1 ml of cold PBS and homogenized.

### Exposure to hyperoxia

Male C57BL/6 mice and cultured macrophages were exposed to hyperoxia as previously described (Patel et al. 2013). Briefly, animals were placed in micro-isolator cages (Allentown Caging Equipment Co., Inc., Allentown, NJ), which were kept in a Plexiglas chamber (BioSpherix, Lacona, NY) and exposed to ≥ 99% O_2_ for up to 48 h. The exposure of murine macrophage-like RAW 264.7 cells was done in sealed, humidified Plexiglas chambers (Billups-Rothenberg, Inc., Del Mar, CA), flushed with 95% O_2_/5% CO_2_ at 37 °C. An oxygen analyzer (MSA; Medical Products, Pittsburgh, PA) was used to monitor the O_2_ concentration in the chamber.

### Western blot analysis

To determine the levels of extracellular HMGB1, RAW 264.7 cells were cultured in serum-free Opti-MEM I medium (Gibco BRL, Grand Island, NY) in 12-well plates and were exposed to either 95% O_2_ alone or 95% O_2_ in presence of GTS-21 for 24 h. After hyperoxic exposure, the levels of HMGB1 in the culture media of treated cells and BAL samples obtained from mice were measured by Western blot analysis. C57BL/6 mice were exposed to ≥ 99% O_2_ for 48 h, followed by inoculation with PA (0.1 × 10^8^ CFUs/mouse), and returned to their cages (21% O_2_) after inoculation. Mice were randomized to receive either GTS-21 or saline, administrated by intraperitoneal injection, every 8 h starting at 32 h during hyperoxia. The levels of NF-κΒ and IκB in the nucleus and cytoplasm of lung cells in these mice were determined in nuclear and cytoplasmic extract prepared by using the NE-PER Nuclear and Cytoplasmic Extraction Reagents kit (Thermo Fisher Scientific, Waltham, MA), according to the manufacturer’s protocol. Fifteen micrograms of nuclear extract, 30 µg of cytoplasmic extract and an equal volume of BAL samples and culture media were loaded on to SDS-polyacrylamide gels (10% and 13%) and then transferred to Immobilon-P membranes (Millipore, Bedford, MA, USA). Non-specific binding sites on the membrane were blocked using 5% nonfat dry milk (Bio-Rad, Hercules, CA) in TBS (Tris-Buffered Saline) containing 1% Tween 20 (TBST) for 1 h at room temperature. The membranes were rinsed three times with TBST and incubated overnight at 4 °C with rabbit anti-HMGB1 polyclonal primary antibody (1:500; Sigma Aldrich), anti-NF-κB p65 rabbit polyclonal antibody (1:500; Santa Cruz Biotechnology, Dallas, TX) and anti-IκB antibody (1:1000; Sigma Aldrich, St Louis, MO) diluted in 5% non-fat dry milk. After three washes in TBST, the membranes were incubated with anti-rabbit horseradish peroxides-coupled secondary antibody (1:5000; GE Healthcare, Piscataway, NJ) for 1 h at room temperature. After washing the membranes thrice in TBST, the immunoreactive proteins were visualized using the enhanced chemiluminescence (ECL) reagent kit (Amersham Pharma Biotech, Piscataway, NJ) as per the manufacturer’s instructions. The image was developed using a UVP Biospectrum 600 Imaging System (Vision Works LS, Upland, CA).

### Immunofluorescence analysis

RAW 264.7 cells were seeded in 12-well plates and allowed to adhere overnight at 37 °C. RAW 264.7 cells were exposed to either 95% O_2_ alone or 95% O_2_ in the presence of GTS-21 for 24 h. After incubation for 24 h, the cells were fixed with 2% phosphate-buffered formaldehyde (pH 7.4) for 15 min and washed three times with PBS. Cells were then permeabilized with 0.2% Triton X-100 (Sigma-Aldrich, St Louis, MO) and nonspecific binding sites were blocked with 10% normal goat serum (NGS) (Chemicon, Temecula, CA) for 20 min. Next, cells were washed with 1% BSA in PBS and incubated with anti-HMGB1 (1:200, Sigma Aldrich, St Louis, MO) or NF-κB p65 (1:200, Santa Cruz Biotechnology, Dallas, TX) primary antibodies overnight at 4 °C. The incubation with the secondary antibody, a goat anti-rabbit immunoglobulin G (IgG) conjugated with Alexa fluor 594 (1:200, Molecular Probes, Eugene, OR) was performed for 1 h. Normal blocking serum without primary antibody was used as a negative control. To visualize the nuclei, cells were counterstained with DAPI (4′,6-diamidino-2-phenylindole). HMGB1 and NF-κB translocation was observed under immunofluorescence microscope (Nikon, Melville, NY).

For quantifying the translocation for NF-κB or HMGB1 signals, fluorescent micrographs obtained from the above assays were subjected to Fiji ImageJ analysis (version 2.0) with the JACoP plugin, which was used to determine a Mander’s Correlation Coefficient using region-of-interest thresholds to measure the magnitude of red signal (staining for NF-κB p65 or HMGB1) colocalizing with the DAPI (staining for the nucleus) signal. The calculated amount of signal colocalization was determined by using the Mander’s Correlation Coefficient that was then represented as a relative amount of NF-κB p65 colocalization with the nucleus or as a percent of HMGB1 located in the nucleus, normalized to the macrophages that remained in room air concentrations of oxygen.

### Phagocytosis assay

The phagocytosis assay was performed as previously described, with minor modifications (Morrow et al. 2007). RAW 264.7 cells were seeded in 48-well plates and were allowed to adhere overnight at 37 °C. RAW 264.7 cells were exposed to either 95% O_2_ alone or 95% O_2_ in the presence of GTS-21 for 24 h. After incubation, RAW 264.7 cells were kept at 37 °C in the presence of FITC-labeled latex minibeads (Polysciences, Warrington, PA), at a ratio of 100:1 (beads/cell). Macrophages were washed with ice-cold PBS to stop phagocytosis, fixed with 4% paraformaldehyde for 10 min and washed with PBS. The fluorescence of the beads that were not phagocytosed by cells was quenched by incubating cells for 10 min with 0.4% trypan blue in PBS. The cytoskeleton was visualized by staining with Texas Red X-phalloidin (Molecular Probes, Eugene, OR) in 1% BSA. Phagocytosis or the uptake of the latex beads was assessed using an immunofluorescence microscope (Nikon Inc, Melville, NY) by counting 250 consecutive individual macrophages/well in duplicates from three independent experiments for each treatment group. Fluorescent beads and cells were manually and blindly counted. The phagocytic activities were quantified using the number of beads per macrophage and presented as percent (%) phagocytic activity compared to RA control group.

### Statistical analysis

The data is presented as the mean ± SEM of at least two independent experiments. The integrated area density of immunoreactive bands was measured using ImageJ Software and the data was analyzed using a Student’s *t* test using MS Excel software. A p-value of < 0.05 was considered statistically significant.

## Results

### The systemic administration of GTS-21 significantly increases bacterial clearance and decreases acute lung injury in a mouse model of VAP

**Fig. 1:**
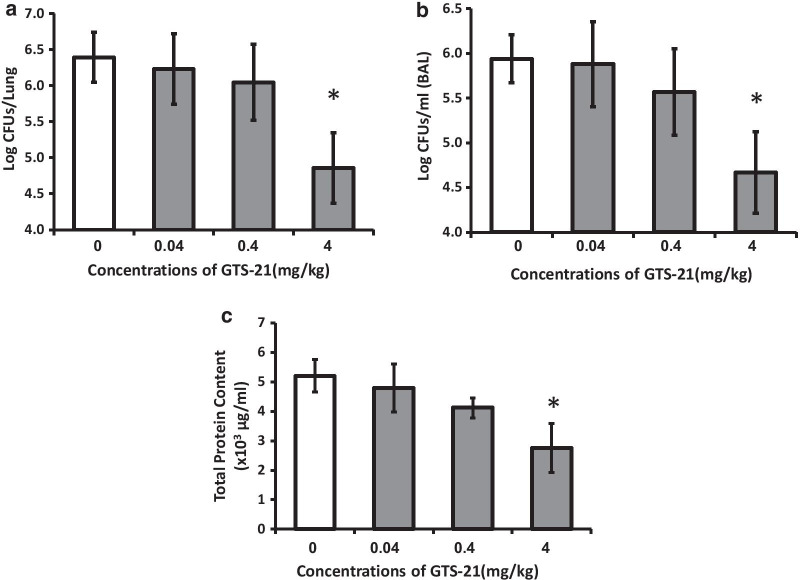
Systemic administration of GTS-21 increases bacterial clearance and decreases acute lung injury in mice exposed to hyperoxia and challenged with PA. C57BL/6 mice were exposed to ≥ 99% O_2_ for 48 h and then inoculated with PA (0.1 × 10^8^ CFUs/mouse), and returned to 21% O_2_ after inoculation. Mice were randomized to receive either GTS-21 (0.04, 0.4, and 4 mg/kg) or saline, administrated by intraperitoneal injection, every 8 h starting at 32 h during hyperoxia. BAL and lung tissue were harvested 18 h after inoculation. Viable bacteria in the airways and lungs were quantified by plating serial dilutions of homogenized lung (**a**) and BAL (**b**), and are expressed as log CFUs/lung and log CFUs/ml of BAL, respectively. The total protein content in the BAL (**c**) was measured as a marker of lung injury. Data represent the means ± SEMs from two independent experiments (n = 5 for control, n = 6 for all GTS-21 treated mice). *p < 0.05, compared with mice receiving normal saline

To determine if GTS-21 increases bacterial clearance under hyperoxic conditions, male C57BL/6 mice were exposed to ≥ 99% O_2_ for 48 h as described previously (Patel et al. 2013) and given either GTS-21 (0.04, 0.4 and 4 mg/kg) or normal saline (control group) intraperitoneally every 8 h, starting at 32 h following the onset of hyperoxic exposure. The mice were then inoculated with PA as described previously (Patel et al. 2013). Bacterial load, both in the airways and lung tissue, was significantly decreased in a dose-dependent manner in animals treated with GTS-21 compared to controls (Fig. 1). GTS-21, at 4 mg/kg, significantly decreased bacterial counts in the lungs (4.85 ± 0.48 log CFUs/lung vs controls 6.39 ± 0.34 log CFUs/lung, p < 0.05; Fig. 1a) and in the airways (4.66 ± 0.45 log CFUs/ml vs controls 5.94 ± 0.26 log CFUs/ml of BAL, p < 0.05; Fig. 1b). Mice that received 4 mg/kg i.p. of GTS-21 also had significantly lower total protein content in the lung lavage samples (a marker of lung injury) compared to mice given normal saline (2755.34 ± 827.78 vs. 5204.70 ± 553.03 µg/ml, p < 0.05; Fig. 1c). These data suggest that GTS-21 is efficacious in increasing bacterial clearance and decreasing acute lung injury in the mouse model of VAP.

### GTS-21 restores hyperoxia-compromised phagocytic activity of macrophages in hyperoxia

**Fig. 2:**
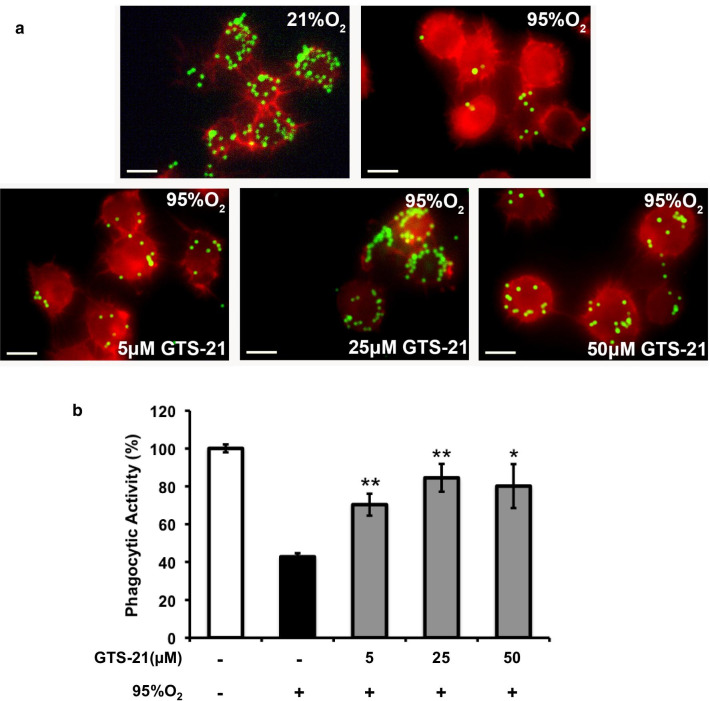
GTS-21 restores hyperoxia-compromised phagocytic activity of macrophages. RAW 264.7 cells were either exposed to 95% O_2_ alone (black bar) or 95% O_2_ in the presence of different concentrations of GTS-21 (grey bars) or remained at 21% O_2_ (white bar) for 24 h. Cells were then incubated with FITC-labeled latex minibeads for 1 h and stained with phalloidin to visualize the cells. **a** Immunofluorescent images of RAW 264.7 cells (Red: actin cytoskeleton; Green: minibeads). At least 250 cells per well from 3 independent experiments were counted and the number of beads per cell was represented as percent beads phagocytosed in the bar graph (**b**). Each value represents mean ± SEM of three independent experiments for each group. *p < 0.05 and **p < 0.01 versus 95% O_2_ alone (black bar). Bar, 10 µm

Previous studies in our lab indicate that hyperoxic exposure can compromise macrophage phagocytic activity and decrease the clearance of pathogenic bacteria (Patel et al. 2013; Entezari et al. 2012). Therefore, we determined if GTS-21-improved bacterial clearance in the mouse model of VAP results from an increase in the phagocytosis of microorganisms by hyperoxic macrophages. As previously reported (Morrow et al. 2007; Patel et al. 2013), the phagocytotic activity of hyperoxia-exposed macrophages was significantly decreased compared to macrophages cultured in room air, 21% O_2_ (42.8 ± 1.9% versus 100.05 ± 2.06, p < 0.001; Fig. 2). In vitro, GTS-21, at 5, 25 and 50 µM, significantly increased the phagocytic activity of macrophages to 70.3 ± 5.8, 84.5 ± 7.3 and 80.1 ± 11.6% respectively, from that of macrophages exposed to 95% O_2_ alone (42.8 ± 1.9%, p < 0.05). These data suggest that GTS-21 can increase hyperoxic macrophage phagocytosis.

### GTS-21 inhibits the accumulation of extracellular HMGB1 induced by hyperoxic exposure

**Fig. 3:**
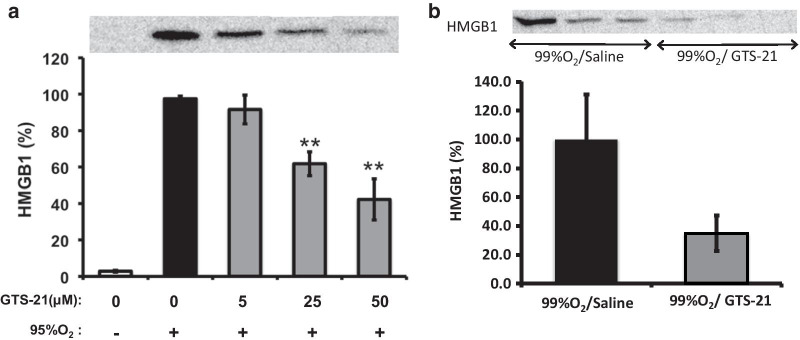
GTS-21 inhibits the accumulation of extracellular HMGB1. **a** RAW 264.7 cells were either exposed to 95% O_2_ alone (black bar) or 95% O_2_ in the presence of a series of concentrations of GTS-21 (grey bars) or remained at 21% O_2_ (white bar) for 24 h. HMGB1 levels in cell culture media were analyzed by Western blot analysis. Representative image of the immunoreactive bands on Western blots is shown. Bar graph shows the integrated density value of HMGB1 bands. The data are expressed as mean ± SEM of three independent experiments. **p < 0.01 versus control 95% O_2_ alone (black bar). **b** C57BL/6 mice were exposed to ≥ 99% O_2_ for 48 h and then inoculated with PA (0.1 × 10^8^ CFUs/mouse), and returned to 21% O_2_ after inoculation. Mice were randomized to receive either GTS-21 or saline, administrated by intraperitoneal injection every 8 h, starting at 32 h during hyperoxia. Representative image of Western blot immunoreactive bands of HMGB1 in the BAL of these mice is shown. Bar graph shows the integrated density value of HMGB1 bands in BAL of mice that received saline (black bar, n = 5) and GTS-21 (4 mg/kg, grey bar, n = 6)

The exposure of macrophages to hyperoxia induces the release of nuclear HMGB1 into the extracellular milieu, which decreases the phagocytic activity of AMs (Liu et al. 2008; Entezari et al. 2012). To determine if the GTS-21-induced increase in host defense and macrophage function is due to a decreased accumulation of extracellular HMGB1, RAW 264.7 cells were exposed to either 95% O_2_ alone or 95% O_2_ in the presence of GTS-21 (5, 25 and 50 µM). Consistent with our previous results (Patel et al. 2013; Entezari et al. 2014), the exposure of RAW 264.7 cells to 95% O_2_ for 24 h induced a significant release of nuclear HMGB1 into the extracellular milieu (Fig. 3a). GTS-21, at 25 and 50 µM, significantly inhibited hyperoxia-induced HMGB1 release (61.76 ± 6.57 and 42.22 ± 11.24 respectively versus 97.43 ± 1.45, p < 0.01; Fig. 3a).

Recent studies in our lab indicated that hyperoxia-suppressed bacterial clearance in PA pneumonia is associated with a substantial accumulation of extracellular HMGB1 in the airways (Patel et al. 2013). To determine if the levels of extracellular HMGB1 in the airways of hyperoxic animals are altered in GTS-21-treated mice, we measured the concentrations of airway HMGB1 in lung lavage fluids from mice exposed to hyperoxia and treated with GTS-21. Figure 3b shows that mice treated with 4 mg/kg i.p. of GTS-21 had a decrease in the accumulation of extracellular HMGB1 in the airways, compared to mice treated with normal saline (34.9 ± 12.23 versus 100.0 ± 31.24, p = 0.106, n = 5 for saline, and n = 6 for GTS-21 treated mice). These data suggest that GTS-21 is efficacious in decreasing the accumulation of high levels of HMGB1 in the airways of hyperoxic animals by decreasing the release of nuclear HMGB1 from lung cells induced by prolonged exposure to hyperoxia.

### GTS-21 inhibits hyperoxia-induced HMGB1 release by blocking HMGB1 translocation from the nucleus to the cytoplasm

**Fig. 4:**
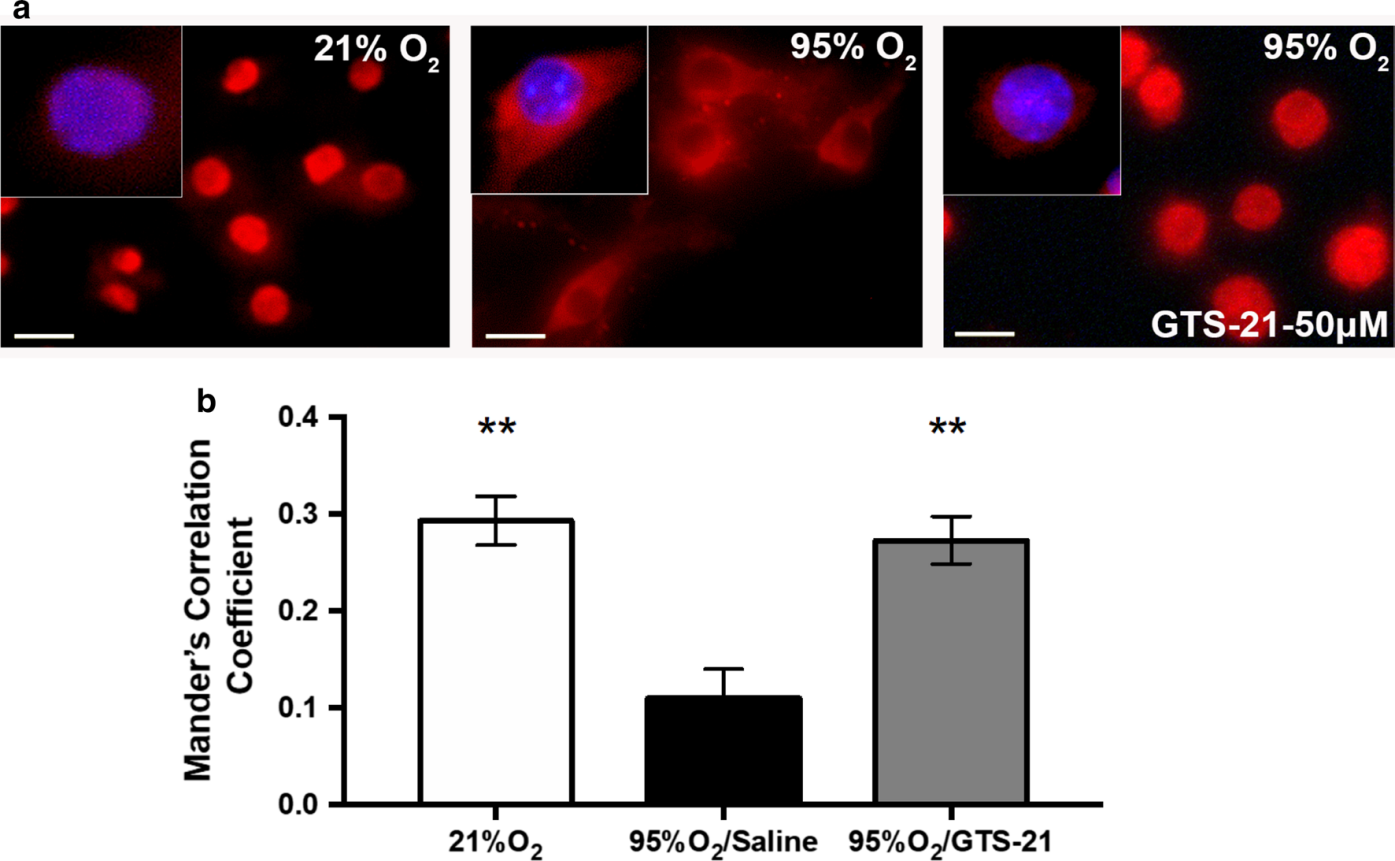
GTS-21 inhibits cytoplasmic translocation of nuclear HMGB1 in macrophages. **a** RAW 264.7 cells were either exposed to 21% O_2_ or 95% O_2_ in the absence or presence of GTS-21 (50 µM) for 24 h. HMGB1 localization was visualized by immunofluorescence microscopy with anti-HMGB1 antibody (Red). Counterstaining with DAPI was used to visualize nuclei (Blue). Bar, 10 µm. Multiple pictures were taken using immunofluorescence microscope to visualize HMGB1. **b** Immunofluorescent micrographs were quantified by determining the amounts of HMGB1 colocalized into the nucleus using a Mander’s correlation coefficient. The immunofluorescence images shown are representative of three independent experiments

Prior to its release, HMGB1 translocates from the nucleus to the cytoplasm, a critical step in its extracellular secretion (Bonaldi 2003; Scaffidi et al. 2002). To test whether GTS-21 can inhibit HMGB1 translocation in hyperoxic macrophages, HMGB1 was visualized using immunofluorescence in RAW 264.7 cells that were exposed to either 95% O_2_ alone or 95% O_2_ in the presence of GTS-21. Exposure to hyperoxia induced the translocation of HMGB1 from the nucleus to the cytoplasm as indicated by a significant decrease in the amount HMGB1 localized within the nucleus (0.109 ± 0.03 Mander’s correlation coefficient, p < 0.01) compared to macrophages that remained in room air conditions (0.292 ± 0.02 Mander’s correlation coefficient; Fig. 4). In contrast, cells incubated with GTS-21 (50 µM) had a significant increase in the amount of HMGB1 located in the nucleus (0.277 ± 0.02 Mander’s correlation coefficient, p < 0.01; Fig. 4) when compared the hyperoxia and vehicle treated cells. The increased levels of HMGB1 in the nuclei of macrophages incubated with GTS-21 were similar to that of the room air control group. This data indicates that GTS-21 is efficacious in inhibiting hyperoxia-induced translocation of HMGB1.

### GTS-21 inhibits hyperoxia-induced NF-κB activation

**Fig. 5:**
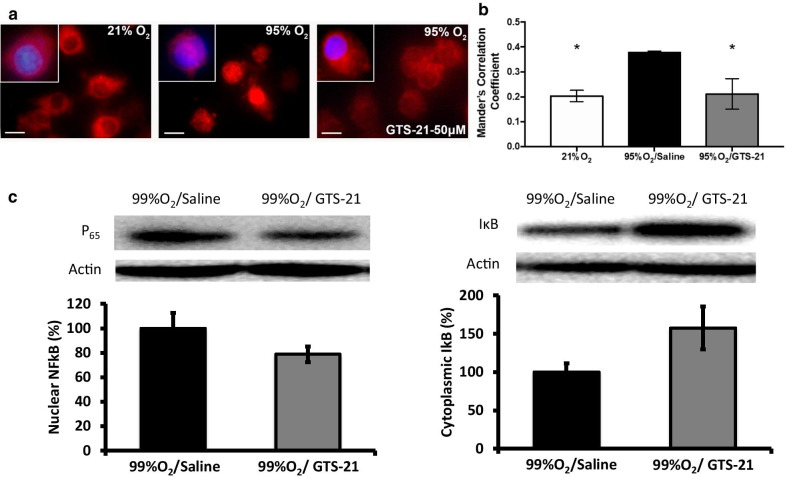
GTS-21 inhibits hyperoxia-induced NF-κB activation. **a** RAW 264.7 cells were exposed either to 95% O_2_ alone or 95% O_2_ in the presence of GTS-21 (50 µM) or remained at 21% O_2_ for 24 h. Following oxygen exposure, cells were washed with PBS, fixed, permeabilized and stained to localize the NF-κB p65 subunit (Red). Multiple pictures were taken using immunofluorescence microscope to visualize the localization of p65 subunit of NF-κB. Counterstaining with DAPI was used to visualize nuclei (Blue). **b** Immunofluorescent micrographs were quantified to determine the amount of p65 translocation into the nucleus by determining their Mander’s correlation coefficient. The immunofluorescence images shown are representative of two independent experiments. **c** C57BL/6 mice were exposed to ≥ 99% O_2_ for 48 h and then inoculated with PA (0.1 × 10^8^ CFUs/mouse), and returned to 21% O_2_ after inoculation. Mice were randomized to receive either GTS-21 (4 mg/kg) or saline, administrated by intraperitoneal injection, every 8 h starting at 32 h during hyperoxia. Lungs of these mice were used to prepare nuclear and cytoplasmic extracts. A representative image of Western blot immunoreactive bands of NF-κB p65 in the nuclear extract and IκB in the cytoplasmic extract of lungs of these mice. Bar graph showing the integrated density value of NF-κB p65 in the nuclear extract and IκB in the cytoplasmic extract of mice that received saline (black bar) and GTS-21 (4 mg/kg) (grey bar; n = 5 for saline, and n = 6 for GTS-21 treated mice). β actin expression was measured as a loading control

To determine the underlying mechanism of the GTS-21-mediated decrease of HMGB1 nucleocytoplasmic translocation, we assessed the localization of NF-κB p65 subunit, a marker for NF-κB activation status (Franek et al. 2002). The p65 subunit of NF-κB was primarily localized in the cytoplasm of RAW cells exposed to room air (0.203 ± 0.023 Mander’s correlation coefficient of nuclear NF-κB p65 subunit) (Fig. 5a, b, 21% O_2_). In contrast, there was an 85.9% increase in the presence of the NF-κB p65 subunit in the nuclei (which is indicative of NF-κB activation) in RAW cells exposed to hyperoxia (0.378 ± 0.006 Mander’s correlation coefficient) (Fig. 5a, b, 95% O_2_). When compared to control macrophages exposed to hyperoxia alone, macrophages incubated with GTS-21 (50 µM) had a significant decreased in nuclear NF-κB p65 (0.211 ± 0.0608 Mander’s correlation coefficient, p < 0.05, Fig. 5a, b), which was not significantly different from cells exposed to room air of a 4.09% increase in NF-κB p65 activation relative to room air control cells. Similarly, a decrease in the levels of nuclear NF-κB p65 subunit was seen in lung cells of mice treated with 4 mg/kg i.p. of GTS-21 (Fig. 5c). In addition, elevated levels of IκB, an inhibitor of NF-κB activation, were found in lung cytoplasmic extracts of mice treated with 4 mg/kg i.p. of GTS-21, compared to mice that received saline (Fig. 5c). These results suggest that GTS-21 is efficacious in blocking hyperoxia-induced NF-κB activation.

## Discussion

We have previously shown that high levels of extracellular HMGB1, released from the nuclei of hyperoxia-exposed lung cells, compromise macrophage phagocytosis and bacterial clearance in a mouse model of VAP (Patel et al. 2013). In this study, our results indicated that GTS-21, an α7nAChR agonist, inhibits hyperoxia-induced accumulation of HMGB1 in the airways of mice in a model of VAP, by attenuating the release of nuclear HMGB1. The inhibition of HMGB1 release was due to a decrease in the translocation of HMGB1 from the nucleus to the cytoplasm. The decrease in HMGB1 translocation was associated with an attenuation of the activation of NF-κB. Importantly, the intraperitoneal administration of GTS-21 dose-dependently increased bacterial clearance from the airways and the lungs and decreased acute lung injury in the mouse model of VAP. Furthermore, hyperoxia-compromised macrophage phagocytosis was restored in cells incubated with GTS-21. These results suggest that GTS-21 increases bacterial clearance by improving hyperoxia-compromised phagocytic function of macrophages by inhibiting HMGB1 translocation and release.

Extracellular HMGB1, released from either the nuclei of intact immune cells or necrotic cells, has been implicated in the pathophysiology of a number of diseases (Scaffidi et al. 2002; Yang et al. 2001). For example, HMGB1 has been postulated to play a role in the pathogenesis of inflammatory diseases such as sepsis and rheumatoid arthritis (Wang 1999; Andersson et al. 2000; Yang et al. 2004; Taniguchi et al. 2003). In addition, extracellular HMGB1 impairs macrophage phagocytosis (Liu et al. 2008; Entezari et al. 2012) and host defense against bacterial infection in mouse models of VAP and CF (Patel et al. 2013; Entezari et al. 2012). Therefore, inhibiting the accumulation of extracellular HMGB1 may significantly attenuate the adverse effects of extracellular HMGB1 in excessive inflammatory responses and compromised innate immunity. In this study, GTS-21 significantly increased bacterial clearance from the lungs of mice exposed to hyperoxia and challenged with PA (Fig. 1). The improved lung function in GTS-21 treated animals with bacterial infection is correlated with a decrease in HMGB1 accumulation in the airways (Fig. 3). Similarly, GTS-21 can inhibit HMGB1 release from immune cells incubated with the inflammatory molecule, LPS (Pavlov et al. 2007; Rosas-Ballina et al. 2009) and decrease serum HMGB1 levels in a mouse model of endotoxemia (Pavlov et al. 2007). In addition, in LPS-stimulated mice, GTS-21 treatment significantly decreased the levels of HMGB1 in the airway and serum (Wang et al. 2019). Nicotine, a non-selective α7nAChR agonist, inhibits HMGB1 release from LPS-stimulated macrophages and increases the survival of animals in a cecal ligation and puncture (CLP) model of sepsis (Wang et al. 2004). Recently, our lab has reported that in mice exposed to 72 h of hyperoxia, 4 mg/kg i.p. administration of GTS-21 significantly attenuates the accumulation of HMGB1 in the airways and the circulation and mitigates inflammatory lung injury by decreasing the infiltration of neutrophils and inflammatory monocytes into the lung and the airways (Sitapara et al. 2020). Thus, these data suggest that the activation of the α7nAChR with agonists, such as GTS-21 or nicotine, may represent a pharmacological approach to combat Gram-negative bacterial infections in organisms subjected to oxidative stress (Entezari et al. 2012; Wang 1999; Ogawa et al. 2006; Rowe et al. 2008).

A critical step in the release of nuclear HMGB1 is its translocation from the nucleus into the cytoplasmic endolysosomes (Li et al. 2003; Rendon-Mitchell et al. 2003). Under normoxic conditions, HMGB1 regularly shuttles between the nucleus and the cytoplasm, but primarily resides in the nucleus (Bonaldi 2003). Here, our results indicated that hyperoxia induces translocation of nuclear HMGB1 into the cytoplasm, which produces accumulation of extracellular HMGB1 in cultured media (Figs. 3, 4).

**Fig. 6:**
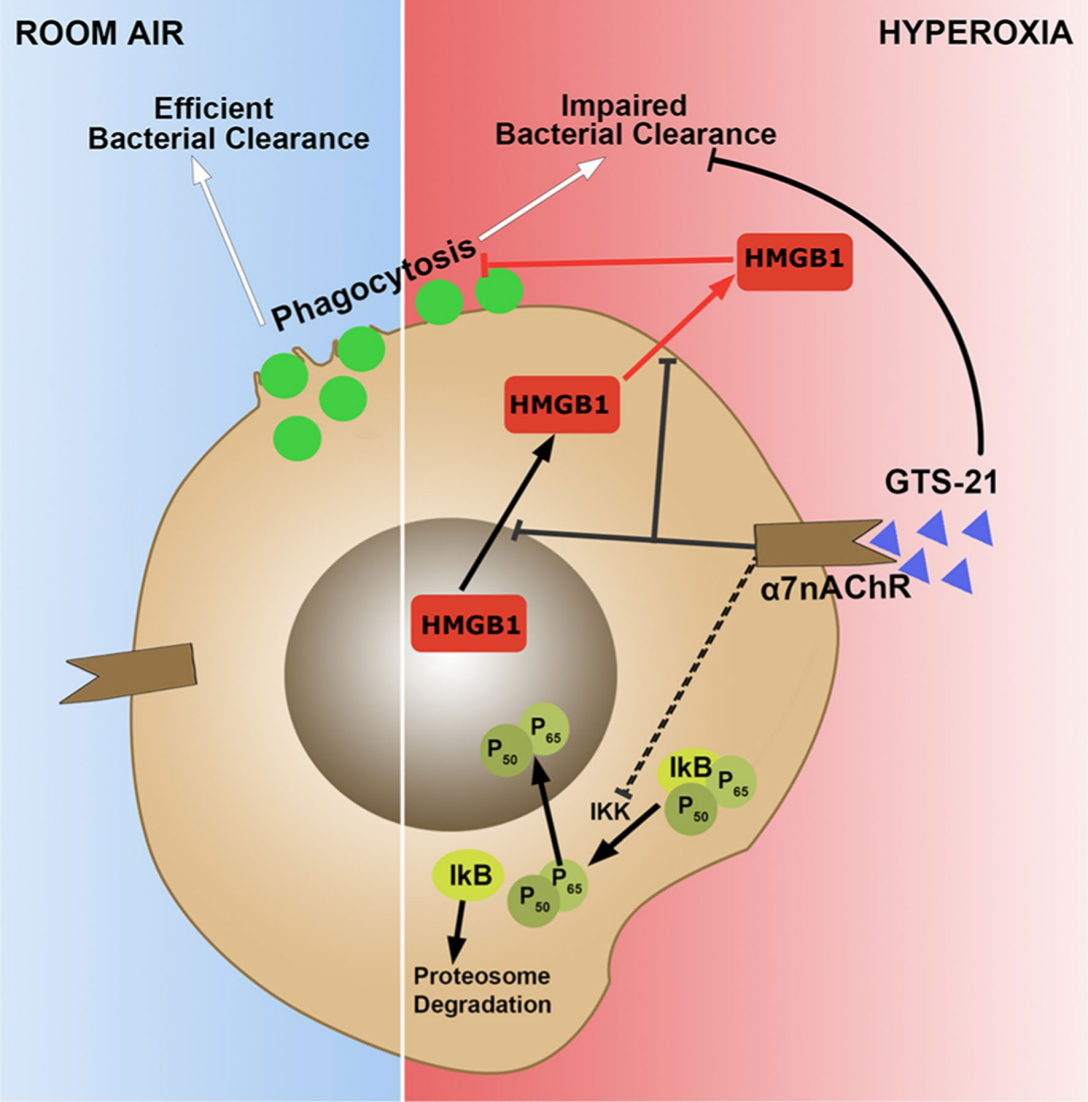
Hypothesized pathway of GTS-21 inhibited hyperoxia-induced HMGB1 release and improved hyperoxia-compromised macrophage function in bacterial clearance. Under room air conditions, alveolar macrophages (AMs) maintain normal phagocytic activity and efficiently clear bacteria. When macrophages are exposed to hyperoxia, NF-κB p65 subunit is translocated into the nucleus, whereas HMGB1 translocates from the nucleus into the cytoplasm and subsequently into the extracellular milieu, decreasing phagocytosis and bacterial clearance by macrophages. GTS-21 significantly attenuates hyperoxia-impaired bacterial clearance by binding to α7nAChR and suppressing HMGB1 translocation into the cytoplasm and release, which subsequently decreases the accumulation of extracellular HMGB1. GTS-21 also inhibits NF-κB p65 subunit translocation into the nucleus, which may prevent HMGB1 translocation and subsequent release into the extracellular milieu

It is possible that GTS-21 suppresses HMGB1 release through a mechanism that resembles vagus nerve stimulation (Wang et al. 2003). Vagus nerve stimulation releases acetylcholine, which acts on the α7nAChR, to inhibit NF-κB signaling and prevent TNF-α production during endotoxemia (Ando 1997; Guarini et al. 2003). As part of the cholinergic anti-inflammatory pathway, the activation of α7nAChR may play a beneficial role in attenuating acute lung injury by decreasing the HMGB1-induced inflammatory response (Andersson 2020). Both nicotine (a non-selective α7nAChR agonist) and GTS-21 inhibit endotoxin-induced NF-κB activation in macrophages (Wang et al. 2003; Pavlov et al. 2007). We have reported that NF-κB activation plays a critical role in hyperoxia-induced HMGB1 release (Wang et al. 2015) and GTS-21 significantly decreases HMGB1 accumulation in the airways and the circulation in mice subjected to hyperoxia (Sitapara et al. 2020). Hyperoxia-induced NF-κB activation in cultured macrophages and mouse lung cells is inhibited by GTS-21, suggesting the possible involvement of NF-κB in mediating GTS-21’s decrease of hyperoxia-induced HMGB1 release (Fig. 6). Overall, our results suggest GTS-21 attenuates the release and accumulation of extracellular HMGB1 by attenuating oxidative stress/infection-induced activation of NF-κB and its down-stream pre-inflammatory responses.

To our knowledge, this study is the first to report that GTS-21 increases macrophage phagocytic function which is essential in combating pulmonary bacterial infections. In this study, GTS-21 significantly increased the phagocytic activity of hyperoxic macrophages (Fig. 2) and increased bacterial clearance from hyperoxia-exposed mice with PA pneumonia (Fig. 1). The restoration of macrophage phagocytosis by GTS-21 occurred at concentrations of 5–50 µM and is, in part, due to the inhibition of HMGB1 release. In addition, extracellular HMGB1 impairs macrophage clearance of apoptotic neutrophils, which may exacerbate bacterial infections by producing inflammatory tissue injury (Liu et al. 2008; Patel et al. 2013). Thus, our results suggest that GTS-21 restores the phagocytic function of macrophages by inhibiting the extracellular accumulation of HMGB1 by attenuating NF-κB activation (Fig. 6). Furthermore, GTS-21 significantly attenuates the levels of pro-inflammatory cytokines, such as TNF-α, and increases the survival of animals subjected to polymicrobial infections (Pavlov et al. 2007). Overall, targeting pathways to attenuate the accumulation of extracellular HMGB1 by GTS-21 may be a novel approach to develop therapies to treat bacterial infections in patients with VAP.

## Conclusions

In summary, our results indicate that the α7nAChR agonist, GTS-21, significantly decreases hyperoxia-induced HMGB1 release from hyperoxic macrophages and lung cells, most likely by inhibiting its translocation into the cytoplasm from nuclei. Importantly, GTS-21 significantly increased bacterial clearance in a mouse model of VAP, most likely by increasing the phagocytotic activity of macrophages due to hyperoxia-suppressed. These results suggest that α7nAChR may represent a pharmacological target for improving the clinical outcome in patients receiving non-invasive and invasive oxygen therapy by augmenting host defense against bacterial infections. These results may also provide mechanistic insight and an avenue for potential treatment strategies in alleviating the pro-inflammatory syndrome and pneumonia commonly found in severe COVID-19 patients.

## Abbreviations

α7nAChR: Alpha 7 nicotinic acetylcholine receptor
BAL: Bronchoalveolar lavage
GTS-21: 3-(2,4 Dimethoxy-benzylidene)-anabaseine dihydrochloride
HMGB1: High mobility group box protein 1
Hyperoxia: Greater than 99% or 95% oxygen
MV: Mechanical ventilation
VAP: Ventilator-associated pneumonia
AM: Alveolar macrophages
LPS: Lipopolysaccharide
PA: *Pseudomonas aeruginosa*
RA: Room air (21% oxygen)
NF-κB: Nuclear factor kappa-light-chain-enhancer of activated B cells
IκB: Inhibitor of NF-κB
TNF: Tumor necrosis factor

## Availability of data and materials

The datasets used and/or analyzed during the current study are available from the corresponding author on reasonable request.
