# Correction to: GTS-21, an α7nAChR agonist, increases pulmonary bacterial clearance in mice by restoring hyperoxia-compromised macrophage function

**DOI:** 10.1186/s10020-021-00357-5

**Published:** 2021-08-23

**Authors:** Ravikumar A. Sitapara, Alex G. Gauthier, Vivek S. Patel, Mosi Lin, Michelle Zur, Charles R. Ashby, Lin L. Mantell

**Affiliations:** 1 Department of Pharmaceutical Sciences, St. John’s University College of Pharmacy and Health Sciences, 8000 Utopia Parkway, Queens, NY 11439 USA; 2 The Feinstein Institute for Medical Research, Northwell Health System, Manhasset, NY 11030 USA

## Correction to: Mol Med (2020) 26:98 https://doi.org/10.1186/s10020-020-00224-9

Following publication of the original article (Sitapara et al. 2020), the author requested to change the title of their re-published paper as follows:

From: The α7 nicotinic acetylcholine receptor agonist GTS-21 improves bacterial clearance in mice by restoring hyperoxia-compromised macrophage function

To: GTS-21, an α7nAChR agonist, increases pulmonary bacterial clearance in mice by restoring hyperoxia-compromised macrophage function

The original article has been corrected.
